# Infertility and cortisol: a systematic review

**DOI:** 10.3389/fendo.2023.1147306

**Published:** 2023-06-29

**Authors:** Bheena Vyshali Karunyam, Abdul Kadir Abdul Karim, Isa Naina Mohamed, Azizah Ugusman, Wael M. Y. Mohamed, Ahmad Mohd Faizal, Muhammad Azrai Abu, Jaya Kumar

**Affiliations:** ^1^Department of Obstetrics and Gynaecology, Faculty of Medicine, Universiti Kebangsaan Malaysia, Kuala Lumpur, Malaysia; ^2^Department of Pharmacology, Faculty of Medicine, Universiti Kebangsaan Malaysia, Kuala Lumpur, Malaysia; ^3^Department of Physiology, Faculty of Medicine, Universiti Kebangsaan Malaysia, Kuala Lumpur, Malaysia; ^4^Basic Medical Science Department, Kulliyyah of Medicine, International Islamic University Malaysia, Kuantan, Malaysia; ^5^Department of Clinical Pharmacology, Faculty of Medicine, Menoufia University, Shebin El-Kom, Egypt

**Keywords:** cortisol, infertility, subfertility, HPA, fertility, pregnancy, stress, sterility

## Abstract

**Introduction:**

Stress and infertility form a complex relationship. In line with this, various stress-related biological markers have been investigated in infertility.

**Methods:**

This systematic review was performed using PRISMA guidelines (i) to report whether cortisol is highly present in infertile patients compared to fertile control; (ii) to report whether there is any significant difference in the cortisol level in infertile subjects that conceive and those that didn’t at the end of assisted reproduction treatments. Original articles involving human (male and female) as subjects were extracted from four electronic databases, including the list of references from the published papers. Sixteen original full-length articles involving male (4), female (11), and both genders (1) were included.

**Results:**

Findings from studies that compared the cortisol level between infertile and fertile subjects indicate that (i) Male: three studies reported elevated cortisol level in infertile patients and one found no significant difference; (ii) Female: four studies reported increased cortisol level in infertile subjects and three studies found no significant difference. Findings from studies that measured the cortisol level from infertile patients that conceived and those that didn’t indicate that (i) Male: one study reported no significant difference; (ii) Female: one study reported elevated cortisol in infertile patients that conceived, whereas two studies reported increased cortisol in infertile patients that was unable to conceive. Five studies found no significant difference between the groups.

**Discussion:**

In the present review we only included the cortisol value that was measured prior to stimulation or IVF treatment or during natural or spontaneous cycles, despite this, there are still variations in the sampling period, assessment techniques and patients’ characteristics. Hence, at present, we are still unable to conclude that cortisol is significantly elevated in infertile patients. We warrant future studies to standardize the time of biological sample collection and other limitations that were addressed in the review to negate the unwanted influencing factors.

## 1 Introduction

Infertility is defined as a disease characterized by failure to conceive after one year or more of regular, unprotected sexual intercourse due to an impaired male or female reproductive system (1). Infertility can be categorized into primary and secondary. Primary infertility is applicable for a woman that has never been diagnosed with a clinical pregnancy and fulfills the criteria for being classified as infertile, whereas a male who was not able to initiate a pregnancy with his female partner and meets the criteria for having infertility is diagnosed to have primary infertility. A woman that unable to conceive once again after previously being diagnosed with a clinical pregnancy and successfully giving birth is known to have secondary infertility. A similar classification is also applicable to males not being able to initiate pregnancy with their female partners but having done so previously (1, 2).

Based on data-driven from 195 countries in the span of 17 years (1990 to 2017), the global burden of infertility has been on the rise, with the age-standardized prevalence rate of infertility increased by 0.37% per annum for females, and 0.29% per annum for males (3). The same study also reported that the age group 35-39 had the highest prevalence rate (3). A wide range of factors has been associated with male and female infertilities including physical problems, lifestyle issues, genetic makeup, psychological problems, and hormonal disorders due to idiopathic reasons (4, 5). The impact of stress-induced psychoendocrinological changes on human reproductive function has been heavily studied over the decades (6, 7), resulting in the discovery of the role of various endogenous hormones including cortisol, catecholamines, vasopressin, gonadotrophins, thyroids, growth hormone, prolactin, and insulin in stress mechanisms (8, 9). Changes in some of these hormones were reported in infertility (10–14). This has led to research questions about whether (i) stress hormones are significantly elevated in infertility (i) causal relationship between elevated stress hormones and infertility, (ii) stress hormones as potential markers of infertility-related risk factors, and (iii) stress hormones as markers of ART outcome prediction.

Cortisol, the primary stress hormone released through the activation of the hypothalamus-pituitary-adrenal (HPA) axis was reported to affect human reproductive function through immunosuppression (15). The effect of cortisol on *in vitro* fertilization (IVF) treatment outcomes was systematically reviewed in the past, and the results were conflicting, with three studies reporting favorable IVF treatment outcomes in the presence of high cortisol levels and five studies reporting low cortisol to positively influence successful outcomes (16). Different treatment cycles during IVF treatment may directly influence the level of cortisol, for instance, hormonal stimulation and invasive procedures-induced stress as well. In the present review, our main aim is to determine the changes in cortisol levels in both male and female infertility in the absence of interference from assisted reproductive technology (ART) treatment procedures. Therefore, for studies that compared the cortisol level between female infertile subjects that conceived and those that didn’t at the end of ART, we only included the cortisol level that was assessed prior to stimulation or induction.

## 2 Methods

### 2.1 Search strategy

The studies were obtained from four online databases including SCOPUS, Web of Science, PubMed, and Ovid MEDLINE from 1946 until September 2022. The last search was formed on 22^nd^ September 2022. The search strategy involved the combination (“AND”) of the following keywords: 1) corti* (cortisol, corticosteroid) OR hydrocorti* (hydrocortisone) OR glucocorti* (glucocorticoid); 2) infertil* (infertility, infertile) OR subfertil* (subfertility, subfertile). In addition, the references of all retrieved articles were reviewed for relevant citations.

### 2.2 Inclusion criteria

All full-length original research articles published in the English language and using humans (male or female or both) as subjects that investigated cortisol and infertility were included. For studies involving female subjects, only studies that recruited infertile subjects, and their biological specimens taken prior to a stimulated cycle or during unstimulated or spontaneous cycles were included. Among these, only studies that compared the differences in cortisol levels between fertile and infertile groups, and the pregnancy outcomes of the infertile subjects were included.

### 2.3 Exclusion criteria

Case series, case studies, books, reviews, letters to the editors, animal studies, cell culture studies, and conference abstracts were excluded. Human studies that specifically looked into infertile subjects with neurological or psychiatric comorbidities were excluded.

### 2.4 Article selection

The articles retrieved from the four databases were independently reviewed by 2 authors (BVK and JK). Any disagreement in the selection process was resolved through discussion to reach a consensus. In general, the articles were screened through three stages. First, articles that did not meet the inclusion criteria were rejected based on their titles. Second, articles that were irrelevant to infertility and cortisol were eliminated based on the abstracts. Third, the remaining articles’ methods, and results were carefully reviewed, and the articles that did not meet the inclusion criteria were eliminated. Reasons for exclusion included (i) if it was not clearly mentioned whether the biological samples to measure cortisol level was taken prior to or after the hormonal stimulation during the infertility treatment, (ii) if the participants were not diagnosed with infertility, (iii) for pre- and post-treatment studies, cortisol level was not assessed prior to treatment, (iv) if the infertile subjects were diagnosed with mood disorders, (v) if the biological samples were taken after induced ovulation, (vi) if the subjects have undergone surgical procedures (varicocelectomy), (vii) infertility patients who were at a stage prior to, during, or after their intrauterine insemination or IVF treatment, (viii) if the biological samples were collected after the embryo transfer, (ix) not reporting absolute cortisol levels.

## 3 Results

**Figure 1:**
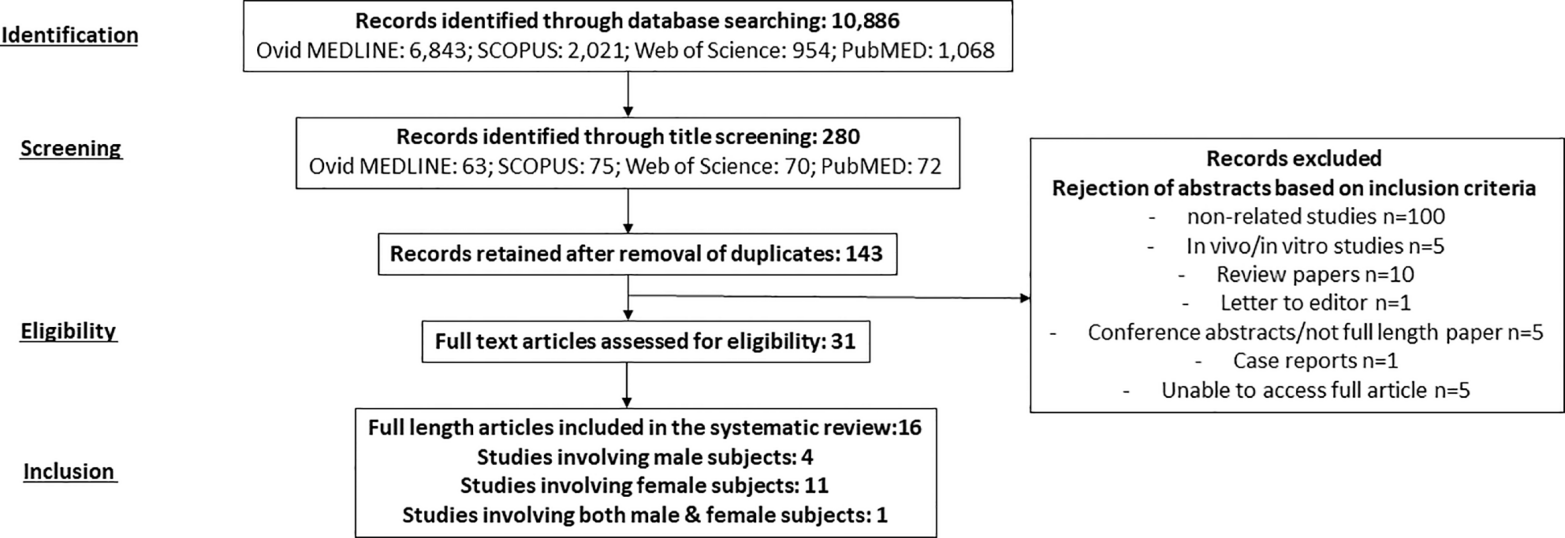
A summary of literature search, screening, and selection of studies based on Preferred Reporting Items for Systematic Reviews and Meta-Analyses (PRISMA) guideline.

Initially, we identified 10,886 articles from four online databases including Ovid MEDLINE (6,843), SCOPUS (2,021), Web of Science (954), and PubMed (1,068). From this, we identified 280 articles through title screening (Ovid MEDLINE: 63, SCOPUS: 75, Web of Science: 70, and PubMeD: 72). Following the removal of duplicates, we found 143 articles. These articles’ abstracts, methods, and results were reviewed based on the inclusion criteria, and this was followed by the rejection of 127 articles. In the end, we included 16 full-length original articles involving males (4), females (11), and both genders (1) as subjects in this systematic review (**Figure 1**).

### 3.1 Study designs and sample characteristics

The study designs that were employed for studies involving male subjects only are case-control prospective studies (17–20), with a total of 400 subjects involving age-matched, healthy fertile controls and infertile subjects. Only one study stated the average age of the participants, infertile (32.4 ± 6.7 years) and fertile (32.7 ± 4.8 to 33.1 ± 5.4 years) (17). Three studies provided the age range of the participants, which was, in general, ranging from 25 to 38 years (18–20) (**Table 1**). One study recruited 150 subjects from both genders, with their age ranging from 30.9 ± 4.9 to 35.3 ± 3.5 years. The study design employed was prospective (21) (**Table 2**).

**Table 1:** Characteristics of the included studies: Male.

References	Title	Study Design	Sample size & Age	Duration of infertility	Inclusion criteria	Cause(s) of infertility	Methods of cortisol measurement	Day, time and types of biological sample collected	Methods & Results	Conclusion
(20)	Impact of emotional disorders on semen quality in men treated for infertility	Case-control prospective study	112 men (60 fertile control vs 52 low fertility) [27 -33 years]	–	Men aged between 27-33, BMI 18.5-24.9, non-smokers, no problem drinking, no history of medications, unable to conceive after 12 months of regular unprotected sexual intercourse with fertile female partner.	None specified	Cortisol level was measured in a standard laboratory.	Serum cortisol collected in the morning	Mean cortisol level was significantly higher in the low fertility group (165.35 µg/dL), compared to the control (130.78 µg/dL) (p < 0.001). Higher Beck Depression Inventory (BDI) was significantly associated with higher cortisol level (r=0.657, p<0.001). Higher State-Trait Anxiety Inventory (STAI-1 and STAI-2) scores were significantly associated with higher cortisol [STAI-1 (r=0.697, p<0.001) STAI-2 (r=0.665, p<0.001)]	Anxiety and depression in subfertile males are associated with increased secretion of prolactin and cortisol.
(17)	Male Fertility: Endocrine Stress Parameters and Coping	Case control prospective study	48 men (14 impaired fertility vs 34 fertile) (average age: Group A: 33.1 ± 5.4, Group B: 32.7 ± 4.8, Group C: 32.4 ± 6.7 to years	–	Male patients aged 18 to 45: Group A: normozoospermia, elevated prolactin (>360 IU/L). Group B: normozoospermia, normal prolactin (<360 IU/L) Group C: impaired fertility, azoospermia or oligozoospermia, or cryptozoospermia	oligozoospermia, cryptozoospermia or azoospermia	Cortisol was assessed using with chemiluminescence immunoassay.	Blood; morning	The cortisol level in group A (612.9 nmol/L) was significantly higher than Group B (478.1 nmol/L) (p<0.05). There was no significant difference between group A+B (557.4 ± 143.4 nmol/L) and group C (580.7± 134.1 nmol/L) in cortisol level. There was no significant difference in depression scores between the groups, based on SVF120 the total of coping strategy was significantly higher in infertile group.	No significant change in cortisol was seen in impaired fertility group, however no comparison was made between Group B and C alone.
(18)	Mucuna pruriens Reduces Stress and Improves the Quality of Semen in Infertile Men	Case-control prospective study	120 men (aged 30-38 years old)	–	Men (30–38 years); healthy control: had previously initiated at least 1 pregnancy and with normal semen profile; infertile group: attending infertility clinic, of same socioeconomic and ethnic status (Indo Aryan) and BMI (19 to 24 kgm2), not on nutritional supplement or vitamins	normozoospermic infertility, Oligozoospermic infertility, asthenozoospermic infertility	Serum cortisol levels were assessed by radioimmunoassay	Venous blood samples; morning (0800h) and evening (1600h)	The morning and evening serum cortisol levels were significantly higher in the infertile groups [morning cortisol ug/dL: normozoospermic (14.1 ± 3.0; P<0.01), oligozoospermic(21.5 ± 0.7; P<0.01) and asthenozoospermic(28.0 ± 1.0; P<0.01)] and [evening cortisol: normozoospermic(10.1 ± 1.6; P < 0.01), oligozoospermic 13.3 ± 4.6 (; P< 0.01) and asthenozoospermic (16.8 ± 1.3; P< 0.01) compared to the healthy control [morning: 10.2 ± 0.2, evening: 5.0 ± 0.6]	The cortisol level was significantly higher in infertile subjects compared to healthy fertile subjects.
(19)	Withania somnifera Improves Semen Quality in Stress-Related Male Fertility	120 total- 60 healthy men, normozoospermic infertile men (20), normozoospermic infertile men under psychological stress (20),normozoospermic infertile men who were cigarette smokers (20) [25-38 years]	Case-control Prospective study	–	Men (aged 25-38 years old) - Control: healthy fertile men who had initiated at least 1 pregnancy and has normal sperm count; Men attending infertility treatment divided into (i) normozoospermic infertile men, (ii) normozoospermic infertile men under psychological stress and (iii) normozoospermic infertile men who were cigarette smokers.	Normozoospermic infertility; infertility of unknown etiology	Serum cortisol was assessed based on method of Foster and Dunn [Foster & Dunn, 1974] (radioimmunoassay)	Blood; collected in the morning (0800h) and evening (1600h)	The mean serum level in the infertile group was higher than the control group in both morning and evening. The cortisol level in healthy fertile group was 10.84 ± 1.63 µg/dL in the morning and 5.8 ± 1.33 µg/dL in the evening.	The cortisol level was significantly higher in infertile subjects compared to healthy fertile subjects.

**Table 2:** Characteristics of the included studies: Male and Female.

References	Title	Study Design	Sample size & Age	Duration of infertility	Inclusion criteria	Cause(s) of infertility	Methods of cortisol measurement	Day, time and types of biological sample collected	Methods & Results	Conclusion
(21)	Stress in couples undergoing assisted reproductive technology	Prospective study	150 patients/75 couples (Mean age, female pregnant: 30.9 ± 4.9, male pregnant: 32.2 ± 4.1, female not pregnant: 33.9 ± 3.9, male not pregnant: 35.3 ± 3.5 years)	From 5.5 ± 3.5 to 5.5 ± 2.4 years	Couples with primary infertility in their first ART treatment cycle, with a BMI of 20.0–29.9 kg/m2.	Primary infertility, female factor: endometriosis, ovulatory disorders, and poor ovarian reserve, and tubal factor Male factor: based on WHO criteria (2010)	Cortisol level was measure using enzyme-linked immunosorbent assay.	Saliva; collected at 15, 30, and 60 min upon waking up prior to the start of gonadotropins in GnRH antagonist cycle (first day of their ART cycle)	There was no significant difference in cortisol levels between different interval upon waking up (15, 30, and 60 mins) in male and female samples. The median value of cortisol level was significantly higher in women who became pregnant than those who did not [24.7 ng/ml (19.9–63.1) vs. 20.7 (10.4–30.4), respectively]. No significant difference was seen in the cortisol level of male between the two groups.	Based on the interval after waking up, the study discovered no statistically significant variation in cortisol levels. However, pregnant women's cortisol levels were higher than those of non-pregnant women, while no such difference was seen in males.

Studies involving female subjects employed designs such as cross-sectional (22, 23), prospective cohort (10), case-control study (24–26), case-control prospective study (7, 27), and prospective study (28–30). The total number of participants recruited was 1102. In general, ten studies reported the average age of the separate groups of patients recruited, such as fertile control (23.5 ± 0.4 to 34 ± 5 years) and infertile group (24.4 ± 0.3 to 33.4 ± 2.3 years) and infertile/pregnant (32.75 ± 5.78 to 36.3 ± 4.76 years) and infertile/non-pregnant (32.94 ± 4.04 to 36.35 ± 3.97 years) (7, 10, 22–29). One study provided the age range of the participants recruited (23-47 years) (30) (**Table 3**).

**Table 3:** Characteristics of the included studies: Female.

References	Title	Study Design	Sample size & Age	Duration of infertility	Inclusion criteria	Cause(s) of infertility	Methods of cortisol measurement	Day, time and types of biological sample collected	Findings	Conclusion/comments
(22)	Endocrine Markers of Fertility Potential in Reproductive Age Women with Idiopathic Hyperprolactinemia (HP)	Cross sectional study	82: 27 healthy women 23.6 ± 0.3 years old, 33 patients with endocrine sub fertility with idiopathic HP (24.4 ± 0.3 years old), and 22 fertile women with idiopathic HP with mean age of 23.5 ± 0.4 years old.	Not stated	Group 0: healthy women who had pregnancy in 2 years back without any gynaecological pathology, without any lactation history Group 1: Infertility for at least two years of unprotected, timed intercourse, and a stable increase of serum prolactin level. Group 2: women with pregnancy 2 years back year but serum prolactin level was increased. The diagnosis of HP occurred before pregnancy and a year after child-birth.	IdiopathicHyperprolactinemia	Serum cortisol was assessed using (“Elisas”) immunoassay analyzer “Ultra Microplate Reader - Elx808”(USA).	Serum; from 8 to 9 am, within the early follicular phase (5-7 days of the menstrual cycle).	Group 0: 475,27 ± 140,59 Group 1 503,84 ± 238,21and Group 2 700,18 ± 352,59nm/l. The cortisol level was higher in the subfertile patients in relation to fertile women in condition of hyperprolactinemia by 37%.	Infertility due to idiopathic hyperprolactinemia elevates serum cortisol level. However, we need to take into account the altered prolactin level in this case, which may disrupt the function of HPA axis, hence the level of cortisol. This may not be the case for the other types of infertility.
(27)	Intensive hormone monitoring in women with unexplained infertility: evidence for subtle abnormalities suggestive of diminished ovarian reserve	Case-control prospective study	24 (12 infertile and 12 healthy control) average age of infertile women 33.2 ± 1.3 years; healthy: 32.8 ± 1.2	4.4 ± 0.8 years	25-40 years old; normal tubal and peritoneal anatomy; 121 ovulatory menstrual cycles from 26 to 32 days; BMI between 18 and 28 (kg/m2>. The male partner had no evidence of male factor infertility.	Unexplained infertility	Serum cortisol level was measured with ELISA using streptavidin technology as the detection system.	blood was taken every other day until the onset of menstruation.	No significant differences in the cortisol level between the groups (P = 0.141), nor was the interaction between group and phase (P = 0.72).	Unexplained infertility had no significant effect on serum cortisol level.
(10)	Interactions of Cortisol and Prolactin with Other Selected Menstrual Cycle Hormones Affecting the Chances of Conception in Infertile Women	prospective cohort study.	305 (205 infertile [26.7 ± 1.9 years] and 100 fertile women [26.8 ± 1.8 years]	Not stated	primary infertility; first entered for infertility therapy, no prior hormone treatment, patent fallopian tubes; no male factors.	Nothing specified	On the third day of the cycle cortisol level was assessed using electrochemiluminescence method using the Cobas c6000 analyzer.	Serum; obtained in the morning (3^rd^ day of the cycle and 7 days after ovulation)	Infertile women recorded higher cortisol compared to the control during each phase of the menstrual cycle (p < 0.001) 3^rd^ day, control: 138.54, infertile: 157.50 ug/dL 7^th^ day after ovulation, control: 143.91, infertile: 216.51 ug/dL. Cortisol level after ovulation in infertile group was negatively correlated with LH during ovulation, progesterone after ovulation, estradiol during and post-ovulation, follicle size, and endometrial thickness during and post-ovulation (r<0, p<0.05). 17 out of 205 infertile women achieved pregnancy (8.3%). Women with + HCG test had significantly lower level of cortisol compared to those with -HCG test result (p < 0.001; 3^rd^ day of the cycle 113.12 versus 161.62 µg/dL; during ovulation 162.24 versus 216.95 µg/dL; after ovulation 163.18 versus 221.46 µg/dL, respectively).	Increased cortisol in infertile women decreased the pre-ovulatory LH peak and estradiol, postovulatory estradiol, affecting the endometrial growth, and conception chances.
(25)	Low 11-deoxycortisol to cortisol conversion reflects extra-adrenal factors in the majority of women with normo-gonadotrophic normo-estrogenic infertility	Case-control study	49 (clomiphene citrate-resistant infertile=26, obese ovulatory control=11, lean ovulatory control=12). Mean age of infertile subjects 33 ± 5 (25 ± 44) obese (34 ± 5), lean 33 ± 4	Not stated	normoestrogenic, normo-gonadotroph, oligomenorrhoeic, witha history of oligomenorrhoea, otherwise healthy and had no endocrine disorders	oligomenhorrea	Cortisol level was assessed using solid-phase 3H radioimmunoassay	Serum; Collected between 0800 and 0900, after 12 h of fasting. Blood collected more than 3 months prior to inclusion in the study.	Comparisons of morning cortisol levels indicated similar morning cortisol concentrations (0.47 ± 0.15, 0.45 ± 0.16 and 0.47 ± 0.18 nmol/l) between 3 groups.	The authors concluded that extra adrenal factors were involved in infertility syndromes studied.
(23)	SIRTI and cortisol in unexplained infertile females; a cross sectional study, in Karachi Pakistan	cross sectional study	135 infertile cases (31.13 ± 5.7) and 207 fertile control (31.22 ± 6.02)	Duration of marriage, 8.03 ± 4.68 for fertile, and 8.47 ± 5.83 years for infertile groups	Infertile: duration of infertility more than two years, subjects aged between 16 and 45 years, from all ethnic backgrounds. Control: healthy females, aged between 16 and 50 years, with a child less than 5 years of age, from all ethnic groups.	Unexplained infertility	Serum cortisol was assessed using enzyme linked immunosorbent assay (ELISA) kits.	Venous blood; collected from 8 till 9 am	The cortisol level in fertile females was 9.98 ± 1.88 (ug/ml), and infertile group was 15.66 ± 7.73(ug/ml), which is significantly higher in the infertile group (<0.01).	In unexplained infertility cortisol level is significantly elevated.
(24)	Hair Cortisol Concentrations as a Biomarker to Predict a Clinical Pregnancy Outcome after an IVF Cycle: A Pilot Feasibility Study	Case-control study	43 subjects with a mean age of 36.3 ± 4.36 years [pregnant: 36.3 ± 4.76, non pregnant: 36.35 ± 3.97]	18.37 ( ± 8.68) in months	BMI of 19–30 kg/m2, no prior fertility treatments, undergoing *in vitro* fertilization with intracytoplasmic sperm injection (IVF/ICSI) or *in vitro* fertilization with preimplantation genetic diagnosis (IVF/PGD), with delayed embryo transfer under the (GnRH) antagonist protocol	Female factor (46.5%), male factor (37.2%), mixed factor (16.2%)	Hair cortisol concentration was measured with a salivary ELISA cortisol kit and expressed in pg/mg	Hair sample was collected on T1: 2nd consultation with reproductive endocrinologist prior to first IVF cycle; T2: 12 weeks following post-transfer visit with the study coordinators (T2), days before human chorionic gonadotropin pregnancy test.	For pregnant women HCC at T1 was 364.63 (571.44) [mean (SD)] and non-pregnant women was 181.06 (169.74) Women with a negative pregnancy test had higher HCC at T2 (181.06 pg/mg vs. 741.06 pg/mg, p < 0.001) but HCC at T2 was not statistically different between pregnant women when compared to non-pregnant women. There were no statistical differences between HCC at T1 and HCC at T2. When BMI, time of infertility, number of follicles and age were included in model as covariates, an interaction effect was noticed between HCC at T1 and T2 F (1, 31) = 0.71, p = 0.01, and η2 = 0.16	HCC might be a promising biomarker to calculate the probability of pregnancy in women using assisted reproductive technologies.
(28)	Pituitary-adrenal and sympathetic nervous system responses to psychiatric disorders in women undergoing *in vitro* fertilization treatment	Prospective study.	264 (The infertile patient that remained non-pregnant, mean age was 33.4 (3.9). vs Infertile that conceived 33.1 (4.1)	7.0 (3.5) years.	Women that came for their first cycle of IVF or ICSI treatment, regular menstrual cycles and no hormonal contraceptive use.	Male factor, female factors, and explained	Cortisol level was measured by radioimmunoassay	Blood samples were taken in the luteal phase before treatment (T1) and on the day of oocyte retrieval (T2) between 8:00 and 9:00 AM.	There is no significant difference in cortisol levels at T1 between the pregnant and nonpregnant women, but at T2, serum cortisol was lower in the conception cycles (P<.001). Serum cortisol in nonpregnant women on T1 was 238.7 (78.2) (nmol/L). Follicular cortisol in the nonpregnant group was significantly higher than the pregnant group (P<.001). Serum cortisol on T2 was significantly correlated with the respective follicular values in all patients (P<.001).	Cortisol concentration imposes negative influence on pregnancy outcome in infertility treatment.
(29)	Prospective study of pregnancy outcome between perceived stress and stress-related hormones	Prospective study	128 (pregnant vs non-pregnant: Mean age ranging from pregnant: 32.75 ± 5.28, non-pregnant: 32.94 ± 4.04)	Not stated	Women aged 40 and below, undergoing the first IVF cycle, capable of completing anxiety and stress test	Primary and secondary	Serum cortisol level was measured through enzyme-linked immunosorbent assay.	Blood samples were collected at 8:00–8:30 on the morning of menstrual cycle day 3	No significant difference was found in cortisol, between the pregnant (18.4 ug/L) and nonpregnant (21.4 ug/L) groups. The cortisol level was significantly related to SDS score.	The authors found significantly higher level of salivary amylase and angiotensin II compared to cortisol in non- pregnant infertile women than the pregnant ones.
(7)	The influence of stress and state anxiety on the outcome of IVF-treatment: Psychological and endocrinological assessment of Swedish women entering IVF-treatment	Case-control prospective study	44 (22 infertile vs 22 fertile controls) infertile (33.4 ± 2.3 years) and fertile (33.1 ± 2.5 years old)	infertility treatment for 3.1 ± 1.6 years and waiting list before treatment was 4.3 ± 1.5 years	Infertile: Primary/secondary infertility, regular menstruation, male partners with normal spermiogram on at least 2 occasions; control: fertile, regular menstruation	Tubal infertility	Serum cortisol level was assessed using DELFIA fluoroimmunoassay kits	Blood samples; collected during a normal natural cycle in the morning between 07.30 h and 08.30 h, on days 3, 10–15 and 19–26.	There was a significant difference in the serum cortisol (p>0.01) during the entire menstrual cycle. There was no significant difference in the plasma cortisol measured between 8–12 o’clock on cycle day 3 between the pregnant and non-pregnant infertile women, and between fertile and infertile women. No significant correlation was reported between the hormonal measure and Karolinska Scales of Personality variables and total STAI scores.	On day 3 of the cycle no significant difference was seen in the cortisol level between the study groups.
(26)	Reproductive problems and intensity of anxiety and depression in women treated for infertility	Case-control study	200 (fertile control 100 and infertile 100) Mean age: infertile: 26.7 ± 1.9 Years, fertile: 26.8 ± 1.8	Not stated	Infertile: Women aged 23−30 with infertility Control: Fertile women with at least two children, and used the mechanical means of contraception during the study.	None specified	Cortisol level was assessed at Diagnostyka Group authorized laboratory	Blood was collected on day of ovulation (without hormonal stimulation)	The serum cortisol concentration was higher in infertile women (212 μg/dl and 217 μg/dl on average) than fertile women (141 μg/dl and 144 μg/dl on average). Cortisol also was higher in non-pregnant women (217 μg/dl and 221 μg/dl on average) than pregnant women (162 μg/dl and 163 μg/dl on average). In infertile women, the cortisol level during and post-ovulation is positively correlated with the severity of trait and state anxiety, symptoms of anxiety and depression.	Emotional state of women undergoing IVF treatment is worse than fertile women.
(30)	Psychological and Hormonal Changes in the Course of in Vitro Fertilization	Prospective study	113	Not stated	Women (age 23-47), completed elementary education, had been previously treated for infertility for at least 2 years, no history of psychological or psychiatric disorders.	Not stated	Cortisol concentration was assessed using the Coat-A-Count and the Double Antibody kits	Baseline measures were taken during early follicular phase (days 3-5 of the cycle), in the morning	The conceived and non-conceived groups recorded normal range of cortisol level at about 15-17 ~µg/dl and remained in this range until the ovum pickup day. On embryo transfer day, a decline in cortisol level was seen in both groups. In luteal phase on expectation of pregnancy results, the cortisol level was increased to 21-22 µg/dl in both groups. Significance correlation was found between cortisol and prolactin level in phase I, III, and IV of IVF treatment in non-conceiving group.	Hormonal and endorphin mediation may play an important role in successful IVF outcome.

The diagnosis or types of infertility specified were diminished ovarian reserve (21), ovulatory disturbance (21), tubal factor (7, 21), unexplained (23, 27), idiopathic hyperprolactinemia (22), oligomenorrhea (25), and endometriosis (21). Some of the studies reported the types of infertility as female factor, male factor, and mixed (24), male factor, female factor, and unexplained (28), primary and secondary (29), normozoospermic infertility (18, 19), oligozoospermia (17, 18), cryptozoospermia (17), azoospermia infertility (17), asthenozoospermia infertility (18), and unknown origin (19). Whereas some studies did not report the diagnosis or types of infertility involved (10, 20, 30) (**Table 4**).

**Table 4:** Types of infertility.

Types of infertility																
Diminished ovarian reserve	Ovulatory Disturbance	Tubal factor	Unexplained	Idiopathic Hyperprolac tinemia	Oligomen horrea	Endomet riosis	Not Stated	Normozoo spermia infertility	Oligozoo Spermia inferility	Cryptozoo spermia	Azoo spermia	Asthenozoo spermia	Unkown origin	female factor, male factor, mixed	male factor, female factor, explained	primary/secondary
(21)	(21)	(7, 21)	(23, 27)	(22)	(25)	(21)	(10, 20, 30)	(18, 19)	(17, 18)	(17)	(17)	(18)	(19)	(24)	(28)	(29)

In general, the infertile females’ mean duration of infertility was ranging from 18.37 months to 8.47 ± 5.83 years. Some studies did not report the duration of infertility (10, 17–20, 22, 25, 29, 30). One of the studies reported the subjects’ duration of the marriage, which was ranging from 8.03 ± 4.68 to 8.47 ± 5.83 years (23). Another study reported the duration of infertility treatment, 3.1 ± 1.6 years, and the period of waiting before the treatment, 4.3 ± 1.5 years (7) (**Table 5**).

**Table 5:** Duration of infertility.

Duration of infertility	
Stated	Not stated
(7) [infertility treatment: 3.1 ± 1.6 years, and waiting list before treatment: 4.3 ± 1.5 years] (21) [5.5 ± 3.5 to 5.5 ± 2.4 years] (23) [duration of marriage: 8.47 ± 5.83 years] (24) [18.37 ± 8.68 months] (27) [4.4 ± 0.8 years] (28) [7.0 (3.5) years]	(10, 17–20, 22, 25, 26, 29, 30)

Some studies recruited newly diagnosed infertile patients (10, 17, 20, 21, 23–25). In some studies, infertile patients have already been exposed to infertility treatment in the past (7, 30). Whereas some studies did not report whether the recruited infertile patients were novices or have prior infertility treatment experience (18, 19, 22, 25–27, 29) (**Table 6**).

**Table 6:** Prior exposure to IVF treatment.

Prior exposure to IVF treatment		
First time IVF	Prior IVF exposure	Not stated
(10, 17, 20, 21, 23, 24, 28)	(7, 30)	(18, 19, 22, 25–27, 29)

### 3.2 Cortisol measurement

Various types of biological samples were collected to assess the cortisol level in the study subjects including serum (7, 10, 18–20, 22, 23, 25–29), saliva (21), and hair (24). Two studies used blood samples but did not specify whether plasma or serum was used for cortisol assessment (17, 30) (**Table 7**).

**Table 7:** Types of biological specimen collected.

Types of biological sample collected			
Blood	Serum	Hair	Saliva
(17, 30)	(7, 10, 18–20, 22, 23, 25, 27–29)	(24)	(21)

Some studies collected the biological specimens in the morning only (7, 10, 17–22, 25, 28, 29), around 0730-0900, upon waking up, and some studies collected samples on multiple time points, such as 15, 30, and 60 minutes after waking up (21). Two studies collected the biological specimens in both morning and evening (18, 19). Whereas some studies did not report the time of biological specimen collection (24, 26, 27) (**Table 8**).

**Table 8:** Time of biological specimen collection.

Biological specimen collection (time)		
Not stated	Morning	Evening
(24, 26, 27)	(7) [0730-0830] (10) (17) (18) [0800] (19) [0800] (20) (21) [15, 30, 60 mins after waking up] (22) [0800-0900] (23) [0800-0900] (25) [0800-0900] (28) [0800-0900] (29) [0800-0830] (30)	(18) [1600] (19) [1600]

The results of cortisol levels were reported in numerous units due to differences in the measuring techniques. Some have reported their results in µg/dL (10, 18–20, 26, 27), nmol/L (7, 17, 28), ng/mL (21), g/dL (30), µg/mL (23), µg/L (29), µmol/L (25), pg/mg (24), and nm/L (22) (**Table 9**).

**Table 9:** Unit of cortisol concentration.

Cortisol concentration (unit)								
µg/dL	nmol/L	ng/ml	g/dL	µg/ml	µg/L	µmol/L	pg/mg	nm/L
(10, 17, 18, 20, 26, 27)	(7, 17, 28)	(21)	(30)	(23)	(29)	(25)	(24)	(22)

Techniques employed in the studies to measure the cortisol level were chemiluminescence immunoassay (10, 17), radioimmunoassay (18, 19, 25, 28, 30), enzyme-linked immunosorbent assay (ELISA) (21, 22, 24, 27), liquid chromatography-mass chromatography (23, 29), and dissociation-enhanced lanthanide fluoroimmunoassay (DELFIA) (7). Two studies did not specify the techniques (20, 26) (**Table 10**).

**Table 10:** Cortisol measurement technique.

Cortisol measurement (technique)					
Not stated	Chemiluminescence Immunoassay	Radioimmunoassay	ELISA	LC-MS	DELFIA fluoroimmunoassay
(20, 26)	(10, 17)	(18, 19, 25, 28, 30)	(21–24, 27)	(29)	(7)

Some studies specified the time of female biological specimen collection as early follicular phase or luteal phase or ovulation phase or prior to the stimulation day (7, 10, 22, 26–30). Some reported the specimens were taken during the time of recruitment or while on the waiting list for treatment or prior to the beginning of treatment (21, 23–25) (**Table 11**).

**Table 11:** Day of biological specimen collection.

Day of biological specimen collection [female subjects]	
Early/mid follicular phase/luteal/ovulatory phase	Prior to the study
(7) [on day 3 of normal natural cycle] (22) [within the early follicular phase, 5-7 days of menstrual cycle] (26) [On the day of ovulation] (27) [every other day until onset of menstruation] Wdowiak et al., 2020 [3^rd^ day of cycle and 7^th^ day after ovulation] (28) [luteal phase before treatment] (29) [Day 3 of menses] (30) [Day 4-5]	(21) [1^st^ day of ART cycle prior to hormonal stimulation] (23) [Blood withdrawn at the time of recruitment] (24) (25) [3 months prior to the study]

### 3.3 Cortisol levels in infertility: male and both genders

A study that assessed the cortisol levels in both male and female patients found no significant difference in the levels of cortisol among males with conceived and non-conceived female partners following the IVF treatment (21) (**Table 12**). Four studies evaluated the cortisol levels in infertile male patients only. Among these, three studies reported elevated cortisol levels among infertile males compared to healthy controls (18–20). One study found no significant difference between infertile and healthy controls (17) (**Table 13**).

**Table 12:** Cortisol level in male infertile patients (Infertile/pregnant vs infertile/non-pregnant).

Infertile/pregnant vs Infertile/non-pregnant (Male cortisol level)		
Infertile/pregnant > infertile/non-pregnant	Infertile/pregnant < infertile/non-pregnant	No significant difference
		(21)

**Table 13:** Cortisol level in male infertile patients (Infertile vs fertile).

Infertile vs fertile (Male cortisol level)		
Infertile > fertile	Fertile > infertile	No significant difference
(18) (19) (20)		(17)

### 3.4 Cortisol levels in infertility: female

Among the studies that recruited female subjects, seven studies compared the cortisol levels between the infertile and fertile female subjects. Out of these, four studies reported elevated cortisol levels in infertile female subjects compared to the fertile group (10, 22, 23, 26). Three studies found no significant difference in the cortisol levels between the fertile and infertile subjects (7, 25, 27) (**Table 14**). Nine studies measured the cortisol levels prior to treatment or stimulation in infertile subjects that conceived at the end of IVF treatment and those that did not. Two studies reported elevated cortisol levels in infertile female subjects that could not conceive following IVF compared to those who could (10, 26). However, five studies found no significant difference in the cortisol levels between the conceived, infertile female subjects and those did not (7, 24, 28–30). In contrast, one study reported that the median value of cortisol was higher among females that conceived compared to those who did not (21) (**Table 15**).

**Table 14:** Cortisol level in female infertile patients (Infertile vs fertile).

Infertile vs fertile (Female cortisol level)		
Infertile > fertile	Fertile > infertile	No significant difference
(10) (22) (23) (26)		(7) (25) (27)

**Table 15:** Cortisol level in female infertile patients (Infertile/pregnant vs infertile/non-pregnant).

Infertile/pregnant vs Infertile/non-pregnant (Female cortisol level)		
Infertile/pregnant > infertile/non-pregnant	Infertile/pregnant < infertile/non-pregnant	No significant difference
(21)	(10) (26)	(7) (24) (28) (29) (30)

## 4 Discussion

### 4.1 Male: infertile vs fertile

Four studies reported changes in blood cortisol levels in healthy fertile and infertile males, with one reporting no significant difference (17), and three found significantly higher cortisol in infertile male patients (18–20). Out of these four studies, only three studies specified the causes of infertility which included normozoospermic, oligozoospermic, cryptozoospermic, azoospermic, asthenozoospermic, and unknown infertility (17, 18). None of the studies reported a significant direct correlation between cortisol levels and infertility characteristics investigated. One study reported higher Beck Depression Inventory (BDI), and State-Trait Anxiety Inventory (STAI-1 and STAI-2) scores to be significantly associated with higher cortisol levels in infertile patients, and BDI score also significantly negatively correlated with sperm count and ejaculate volume. Lower STAI-1 and STAI-2 scores were associated with a higher percentage of sperm with progressive motility (20). A recent systematic review reported mental disorders such as depression, sleep disorders, addiction, eating disorders, and stress negatively impact fertility in males and females (6). It is not within the scope of the present review to relate stress with infertility, nevertheless perceived stress, anxiety, and depression was reported in the past to elevate cortisol level (32, 33).

Harth and Linse (17) found no significant difference in the blood cortisol levels between infertile subjects (normozoospermic, cryptozoospermic, or azoospermic) and a combined cortisol level from Group A (normozoospermic, high prolactin, and stress levels) and Group B (normozoospermic, normal stress and prolactin levels), 580.7 nmol/L (infertile) against 557.4 nmol/L (Group A+B). Nevertheless, earlier reports on prolactin showed that prolactin may directly induce adrenal steroidogenesis, thus increasing the level of cortisol (34, 35). This should be taken into consideration when interpreting the results. In the same study, the cortisol level in Group B subjects was much lower, 478.1 nmol/L, however, no description was given of the individual comparison between group B and C. Harth and Linse (17) recruited participants between the age range of 18-45 years old, a much wider range, whereas the other three studies recruited between the age of 27-33 (20), 30-38 (18), and 25-38 (19) years old participants, with the lowest age being 25 and oldest 38. The average age of the participants recruited by (17) was 32.7 ± 4.8 to 33.1 ± 5.4 years old, whereas the other three studies did not report the participants’ average age. Cortisol is synthesized from cortisone by the enzyme 11β-hydroxysteroid dehydrogenase type 1 (11βBHSD1) and converted to inactive cortisone by 11β-hydroxysteroid dehydrogenase type 2 (11βBHSD2). Age alters the activity of 11βBHSD2 (36). Furthermore, an increase of 17 nmol/L in serum cortisol per year of age was reported. This accounts for approximately 32% of the difference in serum cortisol levels between patients (R2 = 0.315; 36).

Two studies (18, 19) reported the morning cortisol level (10.2 to 10.84 µg/dL) to be higher than the evening cortisol level (5.0 to 5.8 µg/dL) in healthy fertile subjects, which is in line with circadian cortisol rhythmicity reported in the past (37–39). Normozoospermia (18, 19), asthenozoospermia (18), and unknown origin (19) were reported as infertility-related factors in infertile males with higher cortisol. To date, very little literature is available to physiologically link asthenozoospermia to altered cortisol levels. At the preclinical level, asthenozoospermia induced via the inactivation of AMP-activated protein kinase-α1 (AMPKα1) in mice caused no significant changes in plasma cortisol level (40).

### 4.2 Female: infertile vs fertile and infertile/pregnant vs infertile/non-pregnant

Four studies found that the cortisol level in infertile females was significantly higher compared to fertile females (10, 22, 23, 26). Out of this, only one study reported a significant negative correlation between high cortisol (after ovulation) and LH level during ovulation, progesterone level after ovulation, estradiol level during and post-ovulation, follicle size and endometrial thickness during and post-ovulation (10). One study recruited infertile patients with idiopathic hyperprolactinemia hence elevated cortisol levels due to the direct effect of enhanced prolactin on adrenal steroidogenesis should be considered in the interpretation of results (34, 35). Three studies reported no significant difference in the cortisol level (7, 25, 27). All seven studies measured cortisol using serum samples taken in the morning, except for two studies that did not state the time of biological specimen collection (26, 27). In general, studies that reported elevated cortisol levels in infertile females recruited a larger pool of participants (929 subjects in four studies) compared to the studies that reported no significant difference in the cortisol level (117 subjects in three studies). The average age of participants recruited by studies reporting significant differences in the cortisol level (control: 23.6 ± 0.3 to 31.22 ± 6.02; infertile: 24.4 ± 0.3 to 31.13 ± 5.7 years old) is also lower compared to the studies that found no significant difference in the cortisol (control: 32.8 ± 1.2 to 34 ± 5; infertile: 33 ± 4 to 33.4 ± 2.3 years old). Higher age of the control group (in studies that found no significant difference in cortisol level) may have reduced the deficit in the cortisol value between study groups due to the aging-related effects on cortisol value (36, 41).

Two studies reported higher cortisol in infertile/non-pregnant patients (10, 26), however, none of the studies reported a significant association with pregnancy outcomes. Five studies found no significant change in the cortisol level (7, 24, 28–30). Both sets of studies recruited an almost similar pool of participants (525 versus 595 subjects). In general, the average age of participants in studies with elevated cortisol is in their 20s. Infertility factor such as idiopathic hyperprolactinemia (22) was reported in infertile females with elevated cortisol. Hyperandrogenism was reported in PCOS due to dysregulation of 11β-hydroxysteroid dehydrogenase (42, 43).

The menstrual cycle is known to affect the activity of the HPA axis which can result in variation in cortisol synthesis, such as higher cortisol levels during the luteal phase compared to the follicular phase (31). 14 out of 17 selected studies collected the biological specimen for cortisol analysis in the morning, and four studies did not specify the time of collection (10, 17, 20, 30). Physiological cortisol level is usually higher in the morning, especially 30-45 minutes after awakening and gradually reduces for the rest of the day under normal conditions (44).

Eight out of 17 selected studies did not report whether the recruited infertile patients have any prior exposure to IVF treatment procedures. Anticipatory stress was associated with higher stress reactivity for cortisol (45) and higher stress task-induced increase in cortisol (46). Contrary to this, some researchers found no significant association between cortisol awakening response and stress anticipation (47). Furthermore, reports on the effects of stress during and prior to IVF treatment on pregnancy outcomes have been conflicting (48, 49). It will be interesting to see in future studies whether there is a significant difference in stress and stress-related hormones among infertile patients that undergoing ART treatment for the first time and those with prior exposure.

Based on the data we have listed, while high cortisol may have been reported in most of the infertile subjects, this is not always the case (**Tables 13**–**15**). It is challenging to ascribe infertility to cortisol levels due to the presence of various other confounding factors. The complex interactions of multiple hormones, each of which plays a specialized role in regulating fertility, make the human reproductive system even more complex. The stress hormone cortisol interacts with this complex hormonal network and has the potential to cause disruptions (50), which is still poorly understood. Finding the precise mechanisms through which cortisol affects infertility is challenging due to the complexity of the hormonal pathways and feedback mechanisms involved in fertility regulation. Nevertheless, a majority of results have linked chronically elevated cortisol levels with disrupted reproductive endocrinology based on observational and correlational data. For instance, excessive amounts of cortisol might interfere with GnRH’s pulsatile release, which controls the menstrual cycle and ovulation. This can result in irregular or absent ovulation and, ultimately, infertility (51, 52). High cortisol levels could inhibit LH and FSH release as well, which affects ovarian function and lowers the likelihood of pregnancy. The menstrual cycle is hampered by increased secretion of cortisol and prolactin in infertile women by lowering pre-ovulatory LH peak and E2 and post-ovulatory E2 levels that alter endometrial development and thus lower the likelihood of conception (10). However, it’s crucial to remember that some people might be more resilient to the effects of cortisol on reproductive function. In line with this, it was reported that psychopathology, stress, and hair cortisol concentration scores were higher for pregnant women with poorer resilience than for those with better resilience (53). The likelihood that someone would experience infertility due to cortisol can depend on a variety of variables, including genetic differences, general health state, and stress resilience. It is difficult to establish a universal link between cortisol and infertility owing to this individual diversity.

In the present review, we noticed a large span in the age difference of the recruited study participants. The relationship between age and infertility is complex, with aging older having a significant role in both men’s and women’s declining fertility (54–56). In women, the quality and quantity of eggs decrease during aging, resulting in a decreased ovarian reserve (57), which leads to a longer time to conceive, decreased fertility, and a higher risk of pregnancy-related complications (58, 59). An increase in paternal age also has an impact on reproductive outcomes in males, affecting sperm volume, motility, and morphological changes that may result in decreased fertility (55). Individuals’ stress response systems may change as they get older (60), which could have an impact on how cortisol is regulated. Age-related changes in cortisol patterns, such as a diurnal cortisol fluctuation (61, 62), or changes in overall cortisol output (63). These alterations may be caused by aging-related effects on the hypothalamic-pituitary-adrenal (HPA) axis, the system that regulates cortisol. The relationship between cortisol and age might be bidirectional. Aging-related elements including long-term underlying illness (64), endocrinological changes (65), or psychological stressors (66) could affect cortisol levels. On the contrary, cortisol dysregulation by itself may hasten aging (67, 68), resulting in a complicated interplay between cortisol levels and age. While cortisol and age can both have an independent impact on fertility, they may also affect fertility synergistically. The delicate hormonal balance required for fertility can also be upset by altered cortisol levels linked to long-term stress or by age-related changes in cortisol regulation (10, 52). Hormonal imbalances brought on by cortisol may make the age-related decline in fertility worse and aid infertility.

Stress has long been thought to affect fertility as well as other areas of general well-being (69). While stress is associated with elevated cortisol (70), proving a direct cause-and-effect relationship between cortisol and fertility is challenging, and thus it necessitates more research. The delicate hormonal balance involved in reproductive processes is thought to be affected by prolonged exposure to elevated cortisol levels, which may affect fertility. Based on existing literature, in both genders, prolonged stress has been associated with diminished reproductive functions (71, 72). Even though cortisol is part of the stress-fertility relationship, it’s vital to understand that fertility is a complex process that governed by a myriad of factors. Stress affects fertility indirectly through psychological and physiological impacts such as change in lifestyle factors (73, 74), sleep quality (75, 76), and sexual behavior (77). Understanding the connection between cortisol and fertility presents a substantial problem in separating the precise effects of cortisol from the general stress response. Individual variation in stress reactions and coping mechanisms further complicates the interpretation of research findings. While some people can handle stress better than others, some may be more susceptible to its negative consequences (78). Furthermore, patients who are diagnosed as infertile suffer from a great deal of emotional instability as a result of their condition, increasing the risk of anxiety, depression, and other mood disorders substantially. Infertility drugs like leuprolide, gonadotropins, and clomiphene can affect the patient’s psychological well-being causing side effects such as irritability, anxiety, and depression. Hence, it is often challenging to distinguish between the psychological impacts of infertility and the adverse effects of the medications when evaluating symptoms in women receiving infertility-related pharmacotherapy (70). Therefore, it is important to note that stress can cause infertility, and vice versa, and given the complexity, pinpointing cortisol’s exact function in the relationship between stress and fertility is challenging at present.

## 5 Strength and limitations

To our knowledge, this is the first systematic review that investigated the changes in cortisol levels in infertile male and female patients. Prior to this review, Massey et al. (2014) conducted a systematic review of cortisol levels and IVF treatment outcomes and reported three studies to associate higher cortisol with favorable IVF outcomes and five studies to relate lower cortisol to IVF success. The present review aimed to report whether cortisol is highly present in infertile patients compared to fertile healthy subjects, infertile patients who conceived, and those who didn’t at the end of the ART. We only included studies that reported the cortisol level in the unstimulated/natural cycle or prior to hormonal stimulation to rule out changes in cortisol levels due to hormonal stimulation.

One of the limitations we faced in the analysis was variation in the sampling period for studies involving female subjects as the biological specimens were collected on different days of natural cycles such as early follicular phase, day of ovulation, and days after ovulation. Future studies should standardize the time of biological specimen collection.

## 6 Conclusion and future direction

A complex relationship exists between psychological well-being and infertility, where infertility can cause stress to patients through emotional and financial burdens, and mental health disorders such as depression and anxiety could lead to infertility. Biological markers of stress have been extensively investigated and correlated to various health-related problems, including infertility. Seven out of eleven studies that compared the cortisol level between fertile and infertile subjects found significantly higher cortisol in the infertile group. Out of this, only one study correlated cortisol levels with infertility markers. On a separate note, out of 8 studies, only 3 reported significantly higher cortisol levels in infertile subjects that could not conceive at the end of ART. Based on the evidence we gathered through this review, at present, due to variations in study designs, sampling periods, and patients’ characteristics, it is still unclear if high cortisol level causes infertility in males and females. In the present review, we only included the cortisol value that was measured prior to stimulation or IVF treatment, despite this, there are still variations in the sampling period as the collection of biological specimens was carried out during different time phases of the menstrual cycle such as follicular and luteal phases. Hence, we warrant future studies to standardize the time of biological sample collection to negate the unwanted influencing factors.

## Data availability statement

The original contributions presented in the study are included in the article/supplementary material. Further inquiries can be directed to the corresponding authors.

## Author contributions

BK, AK, and JK contributed to conception and design; BK, AK, and JK contributed to data acquisition; BK, AK, and JK were involved in the analysis, interpretation, and drafting of the manuscript; IM, AU, WM, AF and AA revise the manuscript critically for important intellectual content. The version to be published has received final approval from all authors.
